# The effect of iron status on gadolinium deposition in the rat brain: mechanistic implications

**DOI:** 10.3389/ftox.2024.1403031

**Published:** 2024-08-26

**Authors:** John P. Prybylski, Olivia Jastrzemski, Michael Jay

**Affiliations:** ^1^ Pharmacometrics, Pfizer, Groton, CT, United States; ^2^ Molecular Pharmaceutics and Pharmacoengineering, University of North Carolina at Chapel Hill, Chapel Hill, NC, United States; ^3^ University of New Mexico School of Medicine, Albuquerque, NM, United States

**Keywords:** gadolinium, toxicokinetics (TK), iron deficiency, iron overload (IO), MRI

## Abstract

**Introduction:** Sites associated with gadolinium (Gd) deposition in the brain (e.g., the globus pallidus) are known to contain high concentrations of ferric iron. There is considerable debate over the mechanism of Gd deposition in the brain. The role of iron transport mechanisms in Gd deposition has not been determined. Thus, we seek to identify if Gd deposition can be controlled by modifying iron exposure.

**Methods:** Female Sprague-Dawley rats were given diets with controlled iron levels at 2–6 ppm, 6 ppt (20 g/kg Fe carbonyl) or 48 ppm for 3 weeks to induce iron deficiency, overload or normalcy. They were kept on those diets while receiving a cumulative 10 mmol/kg dose of gadodiamide intravenously over 2 weeks, then left to washout gadodiamide for 3 days or 3 weeks before tissues were harvested. Gd concentrations in tissues were analyzed by ICP-MS.

**Results:** There were no significant effect of dietary iron and total Gd concentrations in the organs, but there was a significant effect of iron status on Gd distribution in the brain. For the 3-week washout cohort, there was a non-significant trend of increasing total brain deposition and decreasing dietary iron, and about 4-fold more Gd in the olfactory bulbs of the low iron group compared to the other groups. Significant brain accumulation was observed in the low iron group total brain Gd in the 3-week washout group relative to the 3-day washout group and no accumulation was observed in other tissues. There was a strong negative correlation between femur Gd concentrations and concentrations in other organs when stratifying by dietary iron.

**Discussion:** Gd brain deposition from linear Gd-based contrast agents (GBCAs) are dependent upon iron status, likely through variable transferrin saturation. This iron dependence appears to be associated with redistribution of peripheral deposited Gd (e.g., in the bone) into the brain.

## Introduction

The deposition of gadolinium (Gd) in the brain following Gd-based contrast agent (GBCA) administration has resulted in strict labeling changes for all GBCAs marketed in the United States (U.S. Food and Drug Administration, 2017). Despite attention in the literature since GBCAs were first associated with hyperintensity in the dentate nucleus (Kanda et al., 2014), the mechanism of deposition is still not well understood (McDonald et al., 2018). A leading proposed mechanism involves uptake into the cerebral spinal fluid (CSF) through a glymphatic pathway, which explains the entry of highly polar intact GBCA complexes into the brain by bypassing of the lipoidal blood-brain barrier (Deike-Hofmann et al., 2018); the assumption of this mechanism is that some intact GBCA will simply remain in the brain, slowly releasing Gd, which will redistribute to metal-storage sites in the dentate nuclei, globus pallidus, and thalamus. The clinical significance and consequence of retained Gd in the brain is unknown (Gulani et al., 2017). Currently, there are no studies that have demonstrated a causal link between Gd deposition in the brain and clinical symptoms. However, patient reporting of neurological symptoms post-GBCA administration include headaches, vision changes, and hearing changes (Olchowy et al., 2017). Furthermore, there is evidence of Gd encephalopathy secondary to intrathecal administration and toxicity (Kapoor et al., 2010). The glymphatic pathway mechanism is well-supported by analyses of CSF Gd in both rats and humans (Nehra et al., 2018; Taoka et al., 2018). Support for the CSF-based mechanism has contributed to reduced attention on earlier proposed mechanisms related to iron (Fe) and transferrin (Tf) transport (Prybylski et al., 2016), which has so far only been suggested by the strong correlation of Gd deposition sites and Fe storage sites in the brain (Rasschaert et al., 2018). The possibility that both candidate mechanisms may play a significant role in Gd deposition in the brain has not been explored and could have major implications for clinical risk factors of Gd deposition in the brain.

Fe homeostasis is a complex process involving a myriad of transporters and a family of transport proteins (Duck and Connor, 2016). In plasma, Fe is 98%–100% bound to transferrin, but more than 50% of Fe-binding sites on transferrin are unoccupied. Non-transferrin bound Fe is rapidly taken into peripheral tissues where it can cause oxidative damage (Craven et al., 1987); maintaining low transferrin saturation prevents this outcome and facilitates proper packaging of ferric iron (Fe^3+^) into the storage protein ferritin in tissues. In tissues, Fe turnover is a result of using Fe stores as enzymatic cofactors, but in the brain, Fe is stored in abundance so there is very slow clearance of Fe (Dallman and Spirito, 1977). Gd and ferric Fe share the same charge (3+), but Gd has twice the ionic radius and a higher coordination number. These differences likely explain why Gd only binds a single Fe-binding site on human transferrin and with relatively low affinity (logK_Tf:Gd_ = 6.8, logK_Tf:Fe(III)_ = 21) (Zak and Aisen, 1988). However, there is still a strong correlation between deposition sites for Gd and Fe in the brain in humans and rats (Prybylski et al., 2016; Rasschaert et al., 2018), so it is unlikely that Gd deposition is entirely unrelated to Fe transport and storage mechanisms.

Despite evidence suggesting the importance of Fe in Gd deposition, no studies have been reported to show anything but a correlative relationship. Gd deposition within the dentate nuclei, globus pallidus, and thalamus following multiple GBCA exposures have been confirmed. These iron-rich brain structures are associated with neurodegenerative disorders and manganese accumulation (Swaminathan, 2016). Further, the Fe-Gd correlation is specific and not just a general metal storage error, as no such correlation exists for Cu-Gd or Zn-Gd (Rasschaert et al., 2018). In this study, we sought to determine if there is a causative relationship between iron status and Gd deposition/distribution by using modified iron diets in animals in order to model transient iron deficiency and iron overload. We also examined the impact of time on any Fe-dependent Gd effects.

## Materials and methods

### Animal studies

Female Sprague-Dawley rats (Charles River) were fed standard diets with varying amounts Fe to induce Fe deficiency (-Fe), Fe overload (+Fe) and normal Fe levels (FeC); the concentrations of Fe in the food were 2–6 ppm, 6,000 ppm (20 g/kg Fe carbonyl) and 48 ppm, respectively (Envigo, Madison, WI). Chow for these diets were ordered from a vendor, with the high iron food created by adding sufficient quantity of Fe carbonyl to low-iron chow. These diets were selected based on previous investigations on the effects of Fe and heavy metal deposition (Chua and Morgan, 1996) and iron homeostasis disruptions (Erikson et al., 1997). Water was provided *ad libitum* and cages/food were changed every other day, with weights monitored at the same frequency. The rats were initiated on the study diets 3 weeks before any GBCA was administered to alter Fe status prior to Gd exposure, and they were kept on the diets throughout GBCA dosing and washout. Altered Fe status was assessed in the short-term study prior to GBCA dosing by having total Fe binding capacity and serum Fe measured by the UNC Animal Histopathology and Laboratory Medicine Core.

**FIGURE 1:**
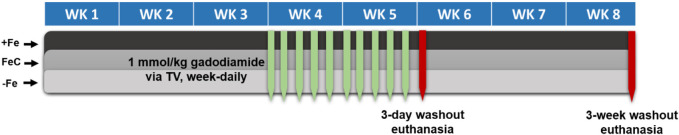
Dosing protocol for iron study. +Fe, FeC and -Fe are the three dietary iron levels for iron overload, normal iron and iron deficiency, respectively. Abbreviations: TV, tail vein; WK, week.

All rats were administered a clinically relevant 1 mmol/kg gadodiamide (a linear, non-ionic GBCA; MedChem Express, Monmouth Junction, NJ) via tail vein, week-daily for 2 weeks (10 mmol/kg cumulative dose; see Figure 1). GBCA dose of 1 mmol/kg gadodiamide was chosen based on Food and Drug Administration (FDA) dosing per surface area recommendation (Medical Imaging Drugs Advisory Committee Meeting, 2017). Additionally, because rodents have a higher clearance rate than humans, doses are often increased to mimic lower clearance (Bruno et al., 2021). After completing 2 weeks of GBCA dosing, rats were either allowed a short-term (3 days) or long-term (3 weeks) washout. There were N = 4 rats per Fe group and N = 12 per washout length (24 total). After the washout period, rats were euthanized by CO_2_ asphyxiation and thoracotomy, then femurs, livers, spleens, kidneys, blood and brains were harvested. The brains were preserved in 15 mL of formalin (Sigma Aldrich, St. Louis, MO) for later sectioning.

### Determination of Gd by ICP-MS

**FIGURE 2:**
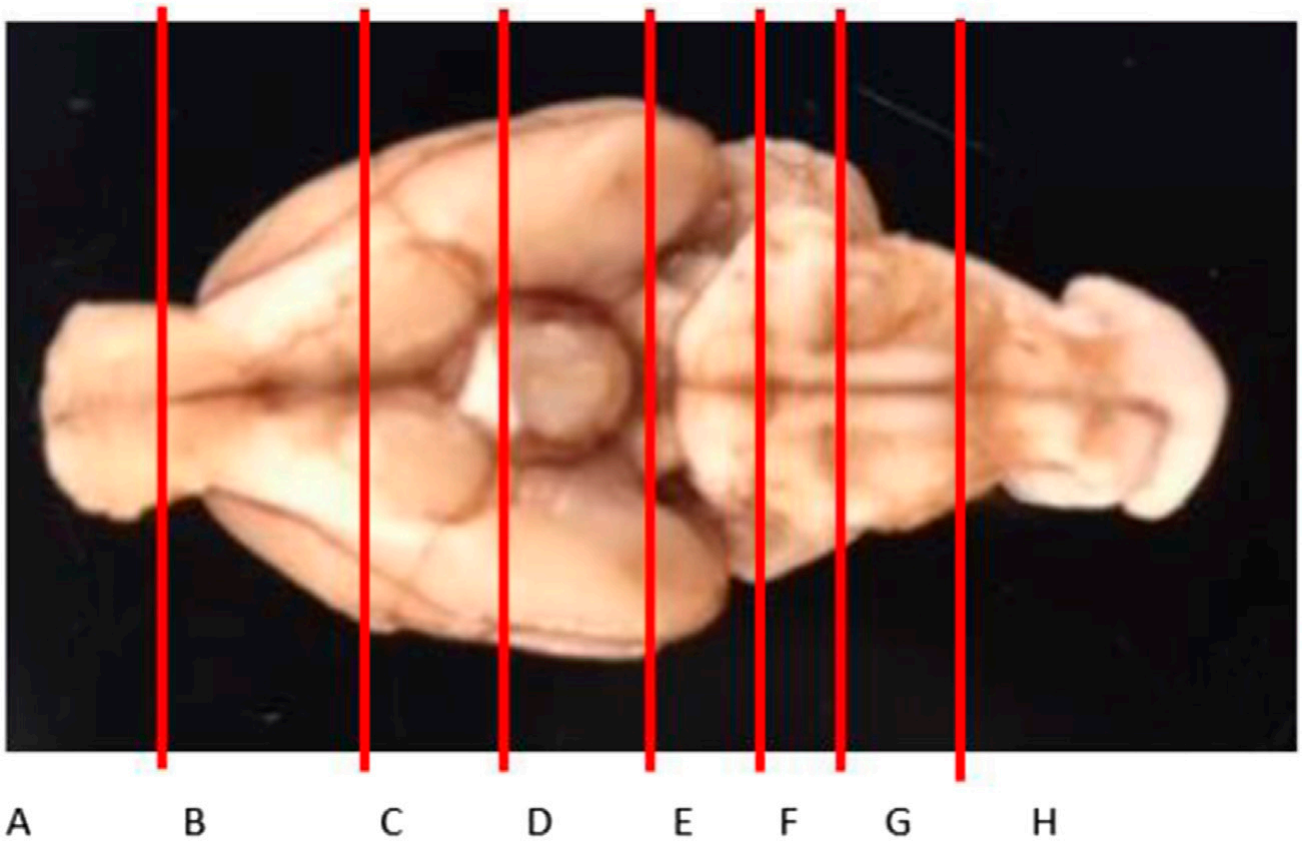
Brain slice map to assess the distribution of Gd in the brain. Slices A and H are technically not parts of the brain (olfactory bulb and the top of the spine, respectively), but are critical to the neuronal system.

Most tissues collected from the rats were weighed and digested in 3 mL of 70% nitric acid (Sigma Aldrich, St. Louis, MO) with no additional processing. The livers were homogenized using a PowerGen™ High-Throughput Homogenizer (Fisher Scientific, Hampton, NH) and triplicate samples of ∼200 mg were used for digestion. The brains were left to set in the formalin for 3–5 days, after which they were manually sliced into 8 sections, as shown in Figure 2; the slices were chosen based on a prior literature report on Gd distribution in the brain (Lohrke et al., 2017), with the addition of the olfactory bulb (A), considered one of the most important parts of the neuronal system in rodents due to its role of smell in rodent evolution, and the top of the spine (H). The slices were weighed and digested in 2–5 mL of 70% nitric acid. Aliquots of each digestion were diluted in ultrapure water to a final nitric acid concentration of 2% and analyzed for Fe and Gd by inductively coupled plasma mass spectrometry (ICP-MS) on an Agilent 7500cx series ICP/MS. The analysis was run in no gas mode. Masses scanned were 156Gd for 200 msec, 56Fe for 200 msec, and the internal standards 45Sc and 115In for 100 msec. The Gd and Fe concentrations in each sample was reported as the mean of 5 replicate scans, and concentrations for sampled homogenates of liver were averaged for each animal.

### Statistical analyses

Within each washout group, organ concentrations of Gd were compared by one-way ANOVA with Sidak-adjusted multiple comparisons. Gadolinium concentrations in different brain slices and comparisons between washout groups were compared by two-way ANOVA with Sidak-adjusted multiple comparisons. Simple linear regression was used to assess the correlations between Fe and Gd concentrations in organs and brain slices. For all analyses, *p*-values or adjusted *p*-values less than 0.05 were considered significant. Data were analyzed using GraphPad Prism 6.05 (San Diego, CA).

## Results

**FIGURE 3:**
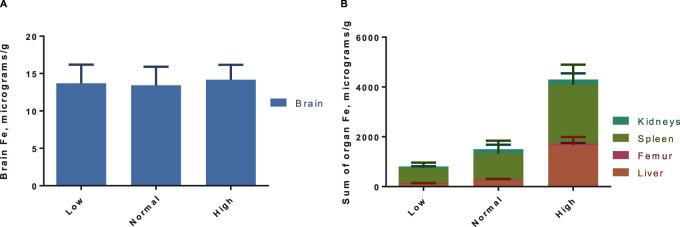
Pooled organ iron concentrations for both gadolinium washout groups (3-day and 3-week post last dose), with total brain **(A)** and rest of the organs in **(B)**. There is a significant trend in increased iron concentrations with increasing dietary iron by two-way ANOVA (*p* < 0.01), which is evident in the non-brain organs **(B)**. Results are pooled because there was no difference in organ Fe across washout groups, except in spleen where there was less Fe in the 3-week washout high iron group (not significant after Sidak adjustment).

**FIGURE 4:**
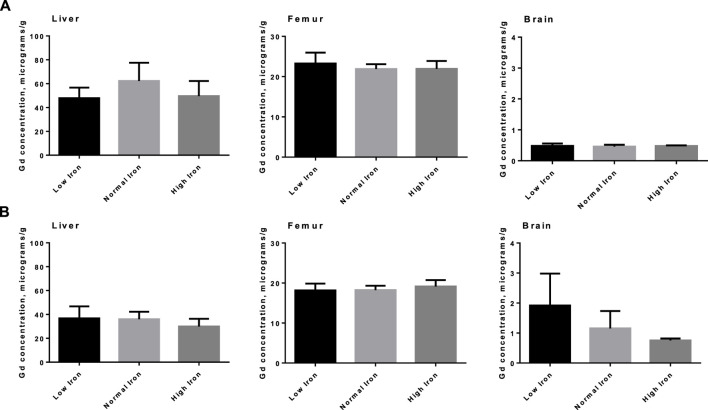
Total Gd organ concentrations in select organs in animals by various amounts of dietary iron. No significant differences were observed in either the 3-day washout group **(A)** or the 3-week washout group **(B)**.

**FIGURE 5:**
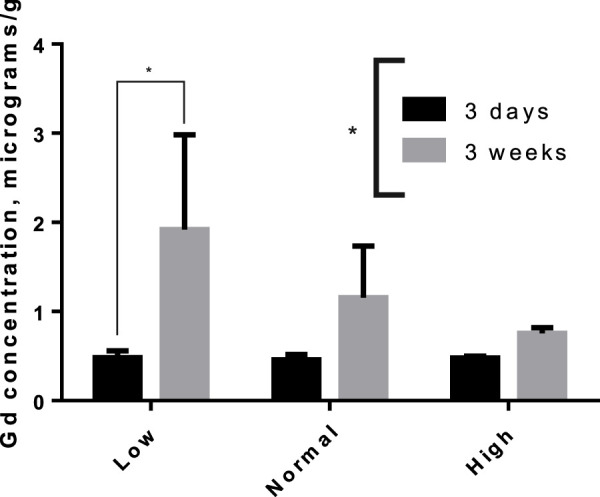
Accumulation of Gd in rat brains between 3-day and 3-week after the last dose of gadodiamide, particularly in the iron deficient group. Asterisks indicated group level significance by one-way ANOVA (asterisk spanning legend) and Sidak-adjusted multiple t-tests (asterisk over connected bars).

The tissue concentrations of Fe (excluding brain) in both washout groups were significantly correlated with level of Fe in the diet (Figure 3), showing that Fe status had been affected by the intervention. There was no difference by between Fe concentrations within diet level when comparing washout groups, except in spleen where the concentration of iron was slightly lower in the 3-week washout of the high iron group (mean ± standard deviation 2049 ± 948 versus 2,670 ± 598 μg/g in high iron 3-week versus 3-day). In the brain, there was no impact of dietary Fe on total brain iron. For all tissues in both washout groups (3-day and 3-week), there was no significant correlation between Gd content and Fe in the diet (Figure 4); the closest to achieving significance was the brain Gd concentration in the 3-week washout group (F = 2.85, *p* = 0.11). In most tissues, the 3-week washout was associated with clearance of Gd relative to the 3-day washout group, but there was significant Gd accumulation in the brain, particularly in the -Fe group (Figure 5).

**FIGURE 6:**
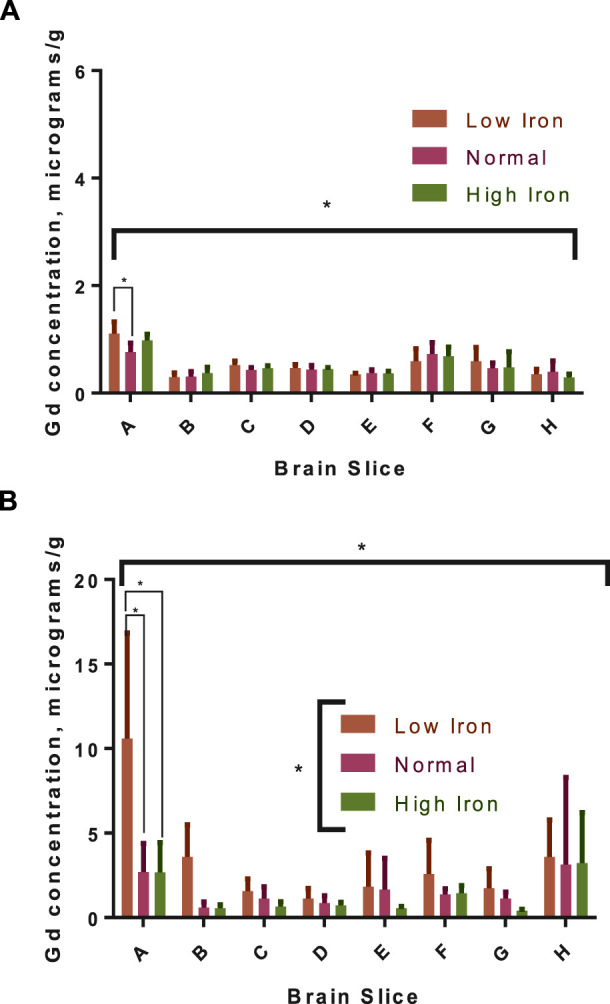
Gadolinium distribution in the brains of rats with different dietary iron. The distribution in the 3-day group **(A)** was generally homogenous, though the concentrations were significantly different across brain slices by two-way ANOVA (indicated by the asterisk under the *x*-axis); diet had a minor, significant effect only in the low iron group compared to normal iron in the olfactory bulb (pairwise, Sidak-adjusted multiple t-tests comparison marked with connecting lines). In the 3-week group **(B)**, there is more Gd in the low iron group for each slice compared to the other two iron groups (group-level difference indicated by asterisk over legend), which is significant in the olfactory bulb (significance of pairwise comparisons indicated); there is also a significant impact of diet on the overall distribution in the slices by two-way ANOVA.

**FIGURE 7:**
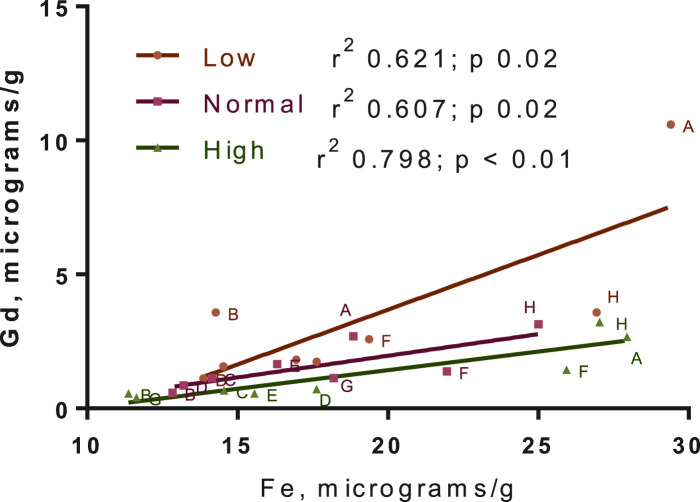
Gadolinium-iron correlation across brain slices. The concentrations here are for the 3-week washout group only, due to blood contamination in 3-day group leading to a flat distribution of iron in that group. Points for all animals are shown. Linear regression best-fit lines are plotted, with r2 and *p*-values for fits in the legend.

The distribution of Gd in the brain was significantly impacted by Fe levels in the diet for the 3-week washout group, but there was no diet-level effect in the 3-day group (Figure 6). Specifically, ∼4-fold higher Gd concentrations were observed in the olfactory bulb for the -Fe group compared to the FeC and +Fe groups (adjusted *p* < 0.0001 for both). The difference in distribution of Gd was significant between the low Fe diet and the normal/high Fe diets. Non-significant trends were observed in other slices, including B (includes a portion of the olfactory bulb) and F (includes the deep cerebellar nuclei), all with more Gd in the -Fe group. In the 3-day group, there was too much blood contamination of the brain to determine the correlation between Fe and Gd concentrations in the brain slices, resulting in a flat distribution of iron across slices. In the 3-week study, the Fe and Gd concentrations in each slice were adequately correlated (r^2^ > 0.6 and *p* < 0.05 for all Fe groups; Figure 7).

Mean tissue Fe concentrations in the 3-week washout group correlated well with mean tissue Gd concentrations, excluding in the whole brain, stratified by diet group and accounting for variability. These correlations were not significant for any tissue in the 3-day washout group. The iron-Gd correlation was strongest in the liver, where the r^2^ was 1.000 with a negative slope, and poorest in the total brain concentration, where the r^2^ was 0.054; kidneys and spleen had r^2^ values of ∼0.6 with negative slopes and femur had an r^2^ of 0.892 with a positive slope. Data for Fe-Gd correlation across tissues is not shown. Groupwise correlations of means (considering variability) were also observed across tissue concentrations of Gd in the 3-week group stratified by iron diet (Table 1). Gd concentrations in the femur had a strong, negative correlation with Gd concentrations in other tissues, though no correlation was significant when adjusted for multiple comparisons.

**TABLE 1:** The correlation matrix for mean gadolinium concentration in each organ in the 3-week washout group stratified by dietary iron levels. The values above the identity line represent the Pearson correlation coefficient, while the values below are the significance of the corresponding correlation, considering variability of mean gadolinium. Femur correlation coefficients are formatted for emphasis; the trend in femur Gd relative to dietary iron is more femur Gd with increasing dietary iron.

	Liver	Femur	Spleen	Kidneys	Brain
Liver		***r = −0.999***	0.718	0.986	0.825
Femur	*p* = 0.024		***−0.691***	***−0.979***	***−0.803***
Spleen	0.490	0.514		0.823	0.986
Kidneys	0.105	0.130	0.385		0.907
Brain	0.382	0.407	0.108	0.277	

## Discussion

The study demonstrated a significant effect of dietary Fe on the long-term retention and distribution of Gd in tissues, particularly the olfactory bulb. GBCAs have demonstrated deposition of Gd in brain regions that are critical for advanced cognition and executive function, behavior and affect, and sensorimotor coordination (Hua et al., 2022). The lack of a diet-level effect on short-term deposition of Gd suggests that at that stage there is no competition with Fe for transferrin, further contributing to evidence that GBCAs deposit in tissues as intact chelate (Robert et al., 2018). However, the long-term results challenge the hypothesis that prolonged Gd retention in a given tissue is caused by release of Gd from GBCAs that had already deposited in that tissue; i.e., we have demonstrated that released Gd re-enters the circulation, as moving from one tissue to another requires re-entering circulation (unless the tissues are in physical contact), and is re-distributed as free Gd ions would be. This is significant in that it suggests a mechanism for the strong correlation between Fe and Gd concentrations in discrete brain structures for less stable linear GBCAs, while the correlation is non-existent for more stable macrocyclic agents (Rasschaert et al., 2018): deposition of the latter is non-specific while current evidence suggests linear agent deposition is primarily by mobilization of peripheral Gd by Fe transport mechanisms. This is further supported by the amount of Gd deposited from linear GBCAs which is bound in large, macromolecular complexes (Gianolio et al., 2017), which may be further non-specific selection of Gd^3+^ for Fe^3+^. However, additional questions are raised about the intracellular distribution of Gd; the divalent metal transport 1 (DMT1) protein is essential for the uptake of transferrin-bound Fe to the brain and it has no cross-reactivity with trivalent ions (Illing et al., 2012), so how would Gd enter the cytoplasm from an endocytic vesicle? The reduction potential for Gd^3+^/Gd^2+^ is much lower than that of Fe^3+^/Fe^2+^, so it is very unlikely that Gd can undergo the same reduction necessary to transport Fe by DMT1. The intracellular distribution of Gd is an important open question about Gd deposition, and one that could be answered with antibody-based methods that have been developed for Fe speciation (Moos and Morgan, 1998).

The olfactory bulb accumulation of Gd observed in this study has not been observed in similar term studies. The accumulation was only significant for the -Fe group, but there was an appearance of higher Gd in the other groups (Figure 5). This is inconsistent with a similar, kinetic study in which rats were given five weekly, high-dose gadodiamide loading sequence, and there was a significant ∼20% reduction in brain Gd in rats euthanized a month after the last dose compared to rats euthanized a week after the last dose (Robert et al., 2018). To be consistent with the present study, the Gd would be expected to slightly increase in the 1-month group. The difference could be attributable to the time lag from the last dose (7 days vs. 3 days), weekly rather than daily GBCA dosing, or the different gadodiamide formulations (Omniscan^®^ vs. gadodiamide without excess ligand). A combination of the time lag and weekly dosing are most likely to have caused the discrepancy as some doses had well over a month to equilibrate in the body, meaning any redistribution of peripheral Gd to the brain (as seen in the Fe study) would have occurred before the first group of rats was euthanized. It is also possible that Omniscan^®^ with its increased stability resulting from excess ligand (Tweedle et al., 1995) has greater early, glymphatic-mediated brain deposition, and the early release of Gd from unformulated Gd results in more peripheral Gd deposition.

The correlations between tissue Fe concentration and tissue Gd concentrations, or between tissue Gd concentrations, further support the conclusion that there is a relationship between Fe and Gd storage. The negative correlation between Gd in femur vs. other tissues suggests that the mechanism behind long-term Gd deposition involves an equilibrium of Gd incorporated into bone and released from bone, similar to calcium; with increased Gd-binding sites on the transferrin in plasma (as Fe levels go from high to low), that equilibrium shifts to more release of Gd from bone, resulting in transferrin-mediated Gd deposition in other tissues. Equilibria may exist in other tissues but since negative correlations are only noted in the femur, the bone turnover equilibrium must be more pronounced than in other tissues. This may have implications for conditions associated with increased bone turnover and patients with risk factors for those conditions. It has already been shown that Gd concentrations are decreased in osteoporotic patients relative to other patients exposed to GBCAs (Darrah et al., 2009); is it possible these patients would have higher amounts of Gd in their liver and brain? These findings also have implications for patients with comorbid Fe deficiency and osteoporosis, or other conditions of increased bone turnover. Since Fe deficiency can induce bone turnover (Toxqui and Vaquero, 2015), there may be a considerable number of patients at risk of excess non-bone Gd deposition.

This study had several limitations, most significantly a lack of additional contrast agents under investigation. If a stable, macrocyclic agent had been used and the same trend was observed with respect to dietary iron, then transferrin-related changes may not impact Gd deposition. It is known, however, that Gd from gadodiamide binds macromolecules *in vivo*, whereas most evidence suggests macrocyclic agents only deposit intact and never release Gd (Rasschaert et al., 2018; Robert et al., 2018). Additionally, several trends identified in the correlation analyses were strong, but not significant in that comparison or in related Fe diet comparisons. This suggests that the study was underpowered to detect those differences, though it was clearly powered sufficiently to detect differences in brain distribution, which was a primary outcome under investigation. Finally, the relatively short washout of 3 weeks for the long-term cohort may not be enough to extrapolate how transient changes in Fe status impact Gd deposition, however a 3 week washout is on par with washout periods used in other animal studies intended to draw comparisons to clinical observations of long-term retention (Davies et al., 2021). This limitation was considered negligible since in the brain kinetic study referenced earlier (Robert et al., 2018), Gd concentrations in the brain had essentially reached plateau levels 1 month after the last dose of gadodiamide. The addition of male rats would have been valuable to the study considering sex differences in Fe handling and bone turnover.

## Conclusion

Fe deficiency increases the concentration of Gd in Fe storage sites of the rat brain, primarily the olfactory bulb, implying that some Gd deposition is the result of Fe transport mechanisms, likely transferrin. The clinical relevance of these findings may extend to patients at risk of Fe deficiency (e.g., in renal failure) and increased bone turnover. Rare earth metal retention in the olfactory neurons and iron homeostasis disruptions via MRI contrast agents could lead to anosmia, which is important for diagnosticians to consider.

## Data Availability

The raw data supporting the conclusions of this article will be made available by the authors, without undue reservation.
